# Ligand-Activated Peroxisome Proliferator-Activated Receptor *β*/*δ* Facilitates Cell Proliferation in Human Cholesteatoma Keratinocytes

**DOI:** 10.1155/2020/8864813

**Published:** 2020-12-22

**Authors:** Chen Zhang, Yang-Wenyi Liu, Zhangcai Chi, Bing Chen

**Affiliations:** ^1^ENT Institute and Department of Otolaryngology, Eye & ENT Hospital, Fudan University, Shanghai 200031, China; ^2^NHC Key Laboratory of Hearing Medicine (Fudan University), Shanghai 200031, China

## Abstract

Cholesteatoma is characterized by both the overgrowth of hyperkeratinized squamous epithelium and bone erosion. However, the exact mechanism underlying the hyperproliferative ability of cholesteatoma remains unknown. In this study, we investigated PPAR *β*/*δ* expression in human surgical specimens of cholesteatoma and analyzed its functional role as a regulator of epithelial keratinocyte hyperproliferation. We found that the expression of PPAR *β*/*δ* was significantly upregulated in cholesteatoma and ligand-activated PPAR *β*/*δ* markedly promoted the proliferation of cholesteatoma keratinocytes. Furthermore, we showed that PPAR *β*/*δ* activation increased PDK1 expression and decreased PTEN generation, which led to increased phosphorylation of AKT and GSK3*β* and increased the expression level of Cyclin D1. Overall, our data suggested that the proliferating effect of PPAR *β*/*δ* on the cholesteatoma keratinocytes was mediated by the positive regulation of the PDK1/PTEN/AKT/GSK3*β*/Cyclin D1 pathway. These findings warranted further investigation of PPAR *β*/*δ* as a therapeutic target for recurrent or residual cholesteatoma.

## 1. Introduction

Cholesteatoma is a benign epidermally derived temporal bone lesion that is locally destructive and frequently recurrent. It is characterized by both the overgrowth of hyperkeratinized squamous epithelium and bone erosion in the middle ear and mastoid cavity. Cholesteatoma causes a myriad of complications including, but not limited to, hearing loss, ossicular erosion, labyrinthine fistula, facial weakness, and intracranial infections. Unfortunately, the molecular events governing cholesteatoma formation are not well established. Nowadays, increasing attention has been paid to the hyperproliferative activity of epithelium, which would play an important role in the pathophysiologic cascade of cholesteatoma [1].

Presently, surgery is the only effective intervention for cholesteatoma. Despite progress in surgical technique, the overall estimated proportion with recurrence 10 years after surgery is more than 70% [2]. It is reported that the high recurrence rate of cholesteatoma is related to cell hyperproliferation [3, 4]. Therefore, we speculate that the hyperproliferative ability of epithelium might play a significant role in the pathogenesis and recurrent pattern of cholesteatoma.

Peroxisome proliferator-activated receptors (PPARs) are members of the nuclear hormone receptor superfamily and include three distinct isoforms, namely, PPAR *α*, PPAR *γ*, and PPAR *β*/*δ* [5]. After binding with specific ligands, PPARs regulate a wide of cellular processes, such as cell proliferation and differentiation, apoptosis, inflammatory responses, and metabolism. During the past decade, the expression of PPAR *γ* has been reported to be upregulated in the cholesteatoma epithelium and to be related to cholesteatoma differentiation [6]. Since PPAR *β*/*δ* is the predominant subtype in human keratinocytes [7] and keratinocytes are the main component of cholesteatoma matrix, it is logical that PPAR *β*/*δ* would also be expressed in cholesteatoma tissues. However, to our knowledge, the potential expression and distribution of PPAR *β*/*δ* in human cholesteatoma has not been investigated.

A number of researches have demonstrated that ligand activation of PPAR *β*/*δ* can induce terminal differentiation of keratinocytes and epithelium [8]. Consistent with these findings, many researchers have also shown that PPAR *β*/*δ* inhibits cell proliferation in epithelium and other cell types, including colonocytes, keratinocytes, cardiomyocytes, fibroblasts, and cancer cell lines [8]. However, the role of PPAR *β*/*δ* in keratinocyte growth remains questioned as there are limited reports demonstrating that ligand-activated PPAR *β*/*δ* can potentiate cell proliferation [9–11]. Di-Poï et al. demonstrated this mechanism by elucidating that the proliferative effect of PPAR *β*/*δ* was mediated through the direct repression of gene expression of phosphatase and tensin homolog deleted on chromosome ten (PTEN) and increase expression of 3-phosphoinositide-dependent-protein kinase 1 (PDK1), which then activated the phosphorylation of protein kinase B (Akt), leading to cell proliferation of keratinocytes [12]. Thus, these evidences suggest that the role of PPAR *β*/*δ* is cell type- and organ-specific.

Previous researches [13] have proved that the PI3K/Akt/PTEN/Cyclin D1 signaling pathway is indeed active in cholesteatoma epithelium and plays a vital role in cholesteatoma keratinocyte hyperproliferation. Since PPAR *β*/*δ* promoted cell proliferation of keratinocytes by modulating PTEN/PDK1/ILK/Akt activity [12], consequently, in this study, we hypothesized that the activation of PI3K/Akt/Cyclin D1 signaling mediated by PPAR *β*/*δ* may be involved in the abnormal hyperproliferation of keratinocytes in cholesteatoma epithelium. Furthermore, given the therapeutic potential of PPAR *β*/*δ* antagonists, further research of its functional role in cholesteatoma is necessary. To test our hypothesis, we investigated the expression and distribution of PPAR *β*/*δ* in middle ear cholesteatoma, elevated the effects of ligand-activated PPAR *β*/*δ*, and explored the mechanisms by which PPAR *β*/*δ* mediated the cell proliferation in the cultured cholesteatoma keratinocytes.

## 2. Materials and Methods

### 2.1. Materials

Highly selective PPAR *β*/*δ* agonist GW0740 and PPAR *β*/*δ* antagonist GSK0660 were purchased from MedChem Express (NJ, United States). All other reagents were obtained from the supplier as indicated and were at least analytical grade. The antibodies used and their sources were also indicated below.

### 2.2. Tissue Preparation and Immunofluorescence

Specimens were obtained from 10 patients (five patients with acquired primary cholesteatoma and five healthy external canal skins) and used for immunofluorescence. Each specimen was fixed in 4% paraformaldehyde for 24 h and then embedded in paraffin. Then, five 5 mm sections were used for immunofluorescence as previously described [14]. The sections were blocked for 1 h in 10% normal goat serum after deparaffinization and rehydration in graded alcohol. After a brief rinse, the sections were incubated overnight with rabbit polyclonal anti-PPAR *β*/*δ* antibody (Genetex, Cambridge, MA, USA) at 250-fold dilution. Subsequently, the sections were incubated with Alexa 555-labeled goat anti-rabbit antibody for 1 hour at room temperature and then stained using blue fluorescent 4′,6-diamidino-2-phenylindole (DAPI). Finally, the sections were examined with an Axioskop microscope (Carl Zeiss, Oberkochen, Germany). This study was approved by the Research Ethics Committee of the Eye and ENT Hospital of Fudan University. Informed consent was obtained from all cholesteatoma patients included in this study.

### 2.3. Cell Culture and Stimuli

Cholesteatoma keratinocytes were isolated and characterized as previously described [14]. In brief, cholesteatoma tissue was obtained and hand carried to the lab after surgical resection. The tissue was then cut into small pieces with scissors and digested with 200 U/ml collagenase IV (Sigma-Aldrich, St. Louis, MO, USA) at 4°C overnight. The digested cells were washed twice with HBSS and then centrifuged at 1,500 rpm for 5 min. The pellet was removed, added to 10 ml keratinocyte serum-free medium (KSFM; Invitrogen, Carlsbad, CA, USA) with 500 units/ml penicillin/streptomycin (Invitrogen, Carlsbad, CA, USA), and cultured in 5% CO_2_ humidified atmosphere at 37°C. The KSFM media and antibiotics were changed every 3 days. Cell cultures between the third and fourth passages were used in this study.

### 2.4. EdU Staining Proliferation Assay

Cell proliferation in response to different treatments was confirmed using EdU imaging kit (Invitrogen, Carlsbad, CA), and analysis was done according to the manufacturer's instructions. Cholesteatoma keratinocytes (5∗10^3^ cells/well) were plated in a Lab-Tech chamber slide (Nalge Nunc International, Cambridge, MA, USA) and grown to 70-80% confluence in KSFM medium, and then treated with control (DMSO), GW0742 (100 nM), or GSK0660 (5 *μ*M) for 24 h. Edu (10 *μ*M) was added 8 h prior to the end of each measurement period. After being fixed for 15 min with 4% paraformaldehyde, the cells were permeabilized with 0.3%Triton X-100 in PBS for 15 min. Then, the cells were incubated with a Click reaction cocktail containing Click reaction buffer, CuSO_4_, Alexa Fluor® 555 azide, and reaction buffer additive for 30 min while protected from light. Next, the cells were incubated with 5 *μ*g/ml Hoechst 33342 for 10 min for DNA staining. Finally, the cells were imaged with fluorescence microscopy, and the percentage of EdU-positive cells was evaluated.

### 2.5. Western Blot Analysis

Cholesteatoma keratinocytes were cultured on 35 mm culture dishes. The cells were grown to 70-80% confluence and then placed in KSFM with control (DMSO), GW0742 (100 nM), or GSK0660 (5 *μ*M). After 24 h of treatment, the cells were washed and isolated using cell lysis buffer (Beyotime Institute of Biotechnology, China) containing protease inhibitors. Equal amounts of total protein were separated on 8% SDS-PAGE and transferred to a PVDF membrane (100 V for 60 min). The membrane was incubated with the primary antibodies overnight at 4°C, followed by the secondary peroxidase-conjugated antibody for 1 h. The bands were visualized by enhanced chemiluminescence and exposure to ECL Hyperfilm (GE Healthcare). The densitometry of bands was quantified with NIH Image 1.63 software. The protein expression was normalized to the amount of beta-actin. The following primary antibodies were used: anti-phospho-PDK1, anti-protein kinase B (AKT), anti-phospho-AKT, anti-phospho-GSK3*β*, and anti-phospho-PTEN (all from Cell Signaling Technology, Danvers, MA, USA). Antibodies against *β*-actin, Cyclin D1, and PPAR *β*/*δ* were from Genetex, Inc. (Genetex, CA, USA).

### 2.6. Statistical Analysis

Statistical analysis was performed using the statistical software package SPSS (Version 11.5). All data were presented as the mean ± standard deviation (M ± SD) and analyzed by the *t*-test. *p* < 0.05 was considered statistically significant.

## 3. Results

### 3.1. Immunolocalization of PPAR *β*/*δ*

**Figure 1:**
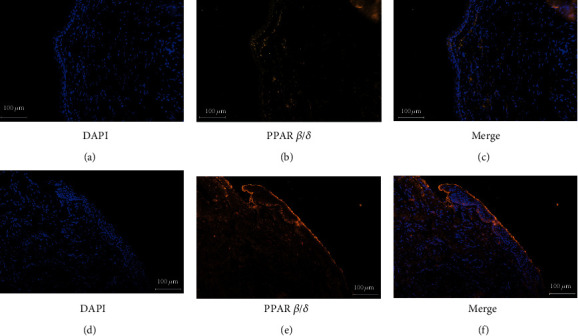
Immunohistochemical staining for proliferator-activated receptor *β*/*δ*. PPAR *β*/*δ* is scantily expressed in external auditory canal skin (a–c). PPAR *β*/*δ* is expressed in the cells mainly in the parabasal and basal layers of cholesteatoma epithelium (d–f). The intensity of its expression is decreased in the granular and prickle cell layers (magnification, ×200).

PPAR *β*/*δ* was distinctly expressed in the nuclei of cells, mainly in basal and parabasal cell layers (Figures 1(d)–1(f)). However, the intensity of its expression was generally weakened in the granular and prickle cell layers (Figures 1(d)–1(f)). In the control skin, scanty staining of PPAR *β*/*δ* was found (Figures 1(a)–1(c)). Immunofluorescent staining for PPAR *β*/*δ* in epithelial tissues of cholesteatoma was consistently stronger than that in control skin.

### 3.2. Cholesteatoma Keratinocyte Proliferation Is Promoted in the Presence of PPAR *β*/*δ*-Selective Agonists

**Figure 2:**
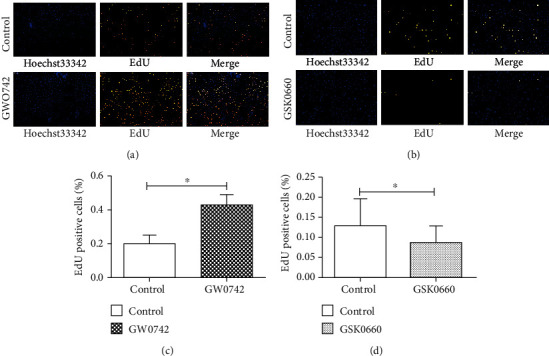
Ligand activation of PPAR *β*/*δ* facilitates the proliferation of human cholesteatoma keratinocytes in vitro. (a, c) The effect of GW0742 (a high affinity PPAR *β*/*δ* agonist) (a) and GSK0660 (a high affinity PPAR *β*/*δ* antagonist) (c) on cell proliferation was detected by EdU assays. (b, d) Data were based on at least three independent experiments and presented as the mean ± SD; ^∗^*p* < 0.05 (vs. control group).

To determine the effect of ligand-activated PPAR *β*/*δ* in cholesteatoma keratinocyte, we treated the cells for 24 h with either GW0742 (a high affinity PPAR *β*/*δ* agonist) or GSK0660 (a high affinity PPAR *β*/*δ* antagonist) and quantified the proliferated cell number following treatment. Because EdU is a thymidine analogue and is incorporated into newly synthesized DNA during S phase, EdU-positive cells are usually the newborn and proliferating cells. As showed in Figure 2, significant increases were observed after 24 h of 100 nM GW0742 treatment in the cholesteatoma keratinocyte (Figures 2(a) and 2(b)). Meanwhile, an antiproliferated effect was observed in cholesteatoma keratinocyte treated with GSK0660, where EdU-positive cell rate decreased significantly following 5 *μ*M GSK0660 treatment (Figures 2(c) and 2(d)). These results suggest that ligand-activated PPAR *β*/*δ* can promote the proliferation of human cholesteatoma keratinocytes.

### 3.3. Activation of PPAR *β*/*δ* by Specific Ligands Increases Expression of PDK1

**Figure 3:**
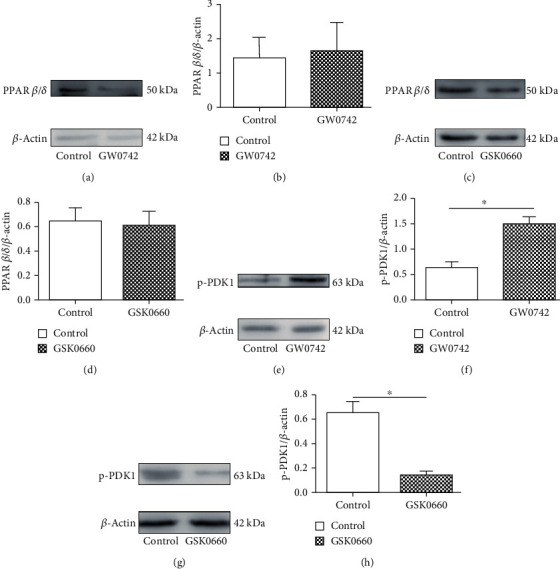
Expression of PPAR *β*/*δ* and ligand activation of target gene (PDK1) in cholesteatoma keratinocytes. (a, c) Expression of PPAR *β*/*δ* was quantified by immunoblot after treating with 100 nM GW0742 (a) or 5 *μ*M GSK0660 (c) for 24 h. (e, g) The effect of PPAR *β*/*δ* agonist (e) or antagonist (g) on expression of the PPAR *β*/*δ*-dependent target gene PDK1 was determined by immunoblot following ligand activation of PPAR *β*/*δ* with 100 nM GW0742 or 5 *μ*M GSK0660 for 24 h. (b, d, f, and h) Data are expressed as the mean ± SD of the mean for experiments run in triplicate. ^∗^*p* < 0.05 (vs. control group).

Cholesteatoma keratinocyte is a transformed keratinocyte cell type with unique biologic behavior that distinguishes it from healthy keratinocytes [15]. PPAR *β*/*δ* is known to be highly expressed in the skin and keratinocytes. To determine whether cholesteatoma keratinocytes express a functional PPAR *β*/*δ*, cells were treated with either 100 nM GW0742 or 5 *μ*M GSK0660. Western blot analysis demonstrated that cholesteatoma keratinocytes constitutively expressed PPAR *β*/*δ* and that GW0742 or GSK0660 had no effect on PPAR *β*/*δ* expression (Figures 3(a)–3(d)). To verify that the proliferation promotion effect of GW0742 is associated with specific ligand activation of PPAR *β*/*δ*, the expression of known and putative PPAR *β*/*δ*-dependent target genes was examined. The expression of the putative PPAR *β*/*δ* target gene PDK1 [13] was significantly induced by GW0742 (Figures 3(e)–3(f)). In addition, treatment with GSK0660, an antagonist of PPAR *β*/*δ*, also significantly reversed the effect of GW0742 on the expression of PDK1 (Figures 3(g)–3(h)). These data show that cholesteatoma keratinocytes are responsive to PPAR *β*/*δ* ligands, as demonstrated by the induction of a known PPAR *β*/*δ*-dependent target genes within 24 h of treatment.

### 3.4. Activation of PPAR *β*/*δ* Promotes the Proliferation of Cholesteatoma Keratinocytes through the PDK1/AKT/GSK-3*β*/Cyclin D1 Pathway

**Figure 4:**
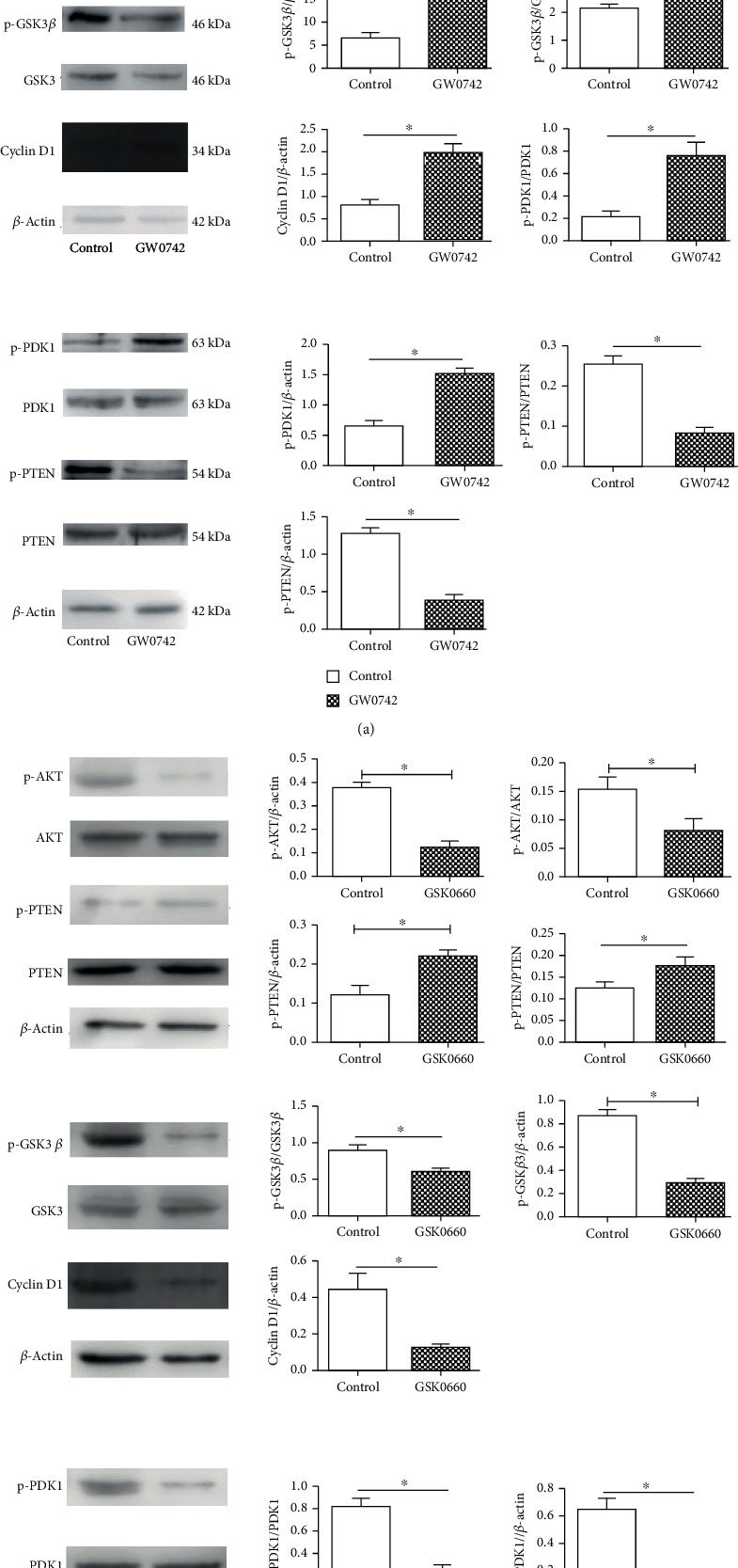
Ligand activation of PPAR *β*/*δ* had effect on PDK1/PTEN/AKT/GSK3*β*/Cyclin D1 signal pathway in cultured cholesteatoma keratinocytes. (a) Western blot showing changes in PDK1/PTEN/AKT/GSK3*β*/Cyclin D1 pathway after treating with 100 nM GW0742 for 24 h. (b) Immunoblots demonstrating the effect of GSK0660 treatment (5 *μ*M, 24 h) on the PDK1/PTEN/AKT/GSK3*β*/Cyclin D1 pathway. *β*-Actin served as loading control. ^∗^*p* < 0.05.

Previous studies suggested that ligand activation of PPAR *β*/*δ* in mouse primary keratinocytes caused antiapoptotic signaling mediated by inhibition of PTEN expression and increased expression of the oncogenes PDK1 and ILK1 leading to increased phosphorylation of Akt [12]. To determine whether this pathway function was similarly in cholesteatoma keratinocytes, we analyzed the expression of PTEN and AKT phosphorylation by means of quantitative western blot analysis. Following treatment with 100 nM GW0742, the cholesteatoma keratinocytes demonstrated increased phosphorylation of AKT and lower expression of PTEN (Figure 4(a)). To fully characterize the signaling pathway, we examined the expression of PTEN, PDK1, AKT, and their downstream targets. The immunoblot (Figure 4(a)) showed that the GW0742 treatment increased the P-AKT (ser473) and its downstream effector Cyclin D1 and inhibited the level of PTEN, with altering phosphorylation activity of GSK3*β* in the cholesteatoma keratinocytes. To determine whether this is a PPAR *β*/*δ*-mediated effect, the specific antagonist of PPAR *β*/*δ*, GSK0660, was used. As shown in Figure 4(b), GSK0660 had the reverse effect on the basal phosphorylation of these kinases. These results indicating that ligand-activated PPAR *β*/*δ* promotes the proliferation of cholesteatoma keratinocytes via upregulation the PDK1/AKT/PTEN/GSK3*β*/Cyclin D1 signaling pathway.

## 4. Discussion

In the present study, we have demonstrated for the first time the localization and elevated expression of the nuclear antigen PPAR *β*/*δ* protein in human middle ear cholesteatoma epithelium. PPAR *β*/*δ* is known to be mainly present in keratinocytes [7] and plays an important role in regulating inflammation, immune responses, cell proliferation, cell differentiation, and apoptosis. In addition, cholesteatoma keratinocyte is a transformed keratinocyte cell type with increased proliferation ability. These results indicated a potential role of PPAR *β*/*δ* overexpression in the pathogenesis of cholesteatoma.

Previously, the PI3K/Akt/Cyclin D1signaling pathway is known to play a crucial role in cholesteatoma epithelial hyperproliferation [13]. The activated PI3K can produce a second messenger PIP3, PIP3 recruits PDK1 and PDK2 to the cell membrane, and then PDK1/PDK2 cooperate to activate Akt completely [13]. In our study, we found that PPAR *β*/*δ* agonist can induce the expression of PDK1, which is served as a putative PPAR *β*/*δ*-dependent target gene [11], significantly affecting the Akt/Cyclin D1 pathway. In addition, PPAR *β*/*δ* activated with highly selective synthetic ligands had an obvious proliferating effect on cholesteatoma keratinocytes. As well as treating the cells with PPAR *β*/*δ* antagonists could reverse this effect. These results lead us to suppose that the PDK1/PTEN/AKT/GSK3*β*/Cyclin D1 pathway is involved in the process of ligand-activated PPAR *β*/*δ*-induced cell proliferation of cholesteatoma keratinocytes. Our work provides a crucial clue for regarding PPAR *β*/*δ* as a potential target to inhibit epidermal keratinocyte proliferation for cholesteatoma therapy.

Another mechanism suggests that PPAR *β*/*δ* promotes cell survival and proliferation via regulation of the PTEN-AKT pathway. As a negative regulator of the PI3K/AKT signaling pathway, the tumor suppressor gene PTEN can regulate cell growth, proliferation, and survival [16]. Previous studies demonstrated that PTEN expression was significantly lower in cholesteatoma epithelium and a significantly inverse correlation between PTEN and p-Akt expressions was found in cholesteatoma [17, 18]. Our study showed decreased PTEN expression in cholesteatoma keratinocytes treated with the PPAR *β*/*δ* agonist GW0742. Although we did not directly determine the effects of activated PPAR *β*/*δ* on PI3K activity, we did detect an increase of phosphorylated PDK1 and AKT, a known downstream target of PI3K. These results suggest that activated PPAR *β*/*δ* decreases PTEN expression and upregulates the AKT signaling pathway to promote proliferation in cholesteatoma keratinocytes.

Glycogen synthase kinase-3*β* (GSK3*β*) has been found to be involved in a variety of cellular processes, such as metabolism, differentiation, and apoptosis [19]. As the Akt substrate, GSK3*β* can be phosphorylated and inactivated by all three isoforms of Akt and is negatively regulated by Akt activity [20, 21]. In our study, GW0742 treatment enhanced the phosphorylation of Akt (Ser473) and GSK3*β* (Ser9) by activating the PDK1/AKT/GSK3*β* signaling pathway. Once GSK3*β* is phosphorylated, the degradation of Cyclin D1 induced by GSK3*β* would be inhibited. Because of the inhibition of PTEN and the activation of AKT, Cyclin D1 would also be induced and its expression level increased. Together, these results may partly explain the mechanism by which the PPAR *β*/*δ*-selective agonists cause proliferation in cholesteatoma keratinocytes.

Based on the findings described above, we propose a model for the role of PPAR *β*/*δ* in regulating cell proliferation in cholesteatoma keratinocytes. After activation of PPAR *β*/*δ*, increased PDK1 expression and decreased PTEN generation lead to increased phosphorylation of AKT and GSK3*β*. These changes increase the expression level of Cyclin D1, which, in turn, promotes the cell cycle transition from G1 to S phase. Taken together, our findings indicate that PPAR *β*/*δ* activation promotes cell proliferation in cholesteatoma keratinocytes through the regulation of the PDK1/AKT/PTEN/GSK3*β*/Cyclin D1 pathway.

In summary, we demonstrated that ligand activation of PPAR *β*/*δ* regulates cell proliferation in cholesteatoma keratinocytes and that upregulation of PDK1 and modulation of the AKT/PTEN/GSK3*β*/Cyclin D1 pathway may be involved. These findings have important implications not only for understanding the molecular mechanism of PPAR *β*/*δ* in cholesteatoma but also by providing novel insights into the treatment of recurrent or residual cholesteatoma.

## Data Availability

The data used or analyzed during the current study are available from the corresponding author on a reasonable request.
